# Braithwaite’s Retrospect

**Published:** 1846-05-01

**Authors:** 


*Braithwaite's Retrospect.*—The 12th number of this useful practical compendium of periodical literature comes to us punctually from the press of Daniel Adee, Esq., of New York city. We have seen somewhere a comparison of this with the work last mentioned, which gives to the latter the preference as regards the *reports;* but attributes to the former the best selections. We need not express our opinion on this point, as each is so valuable and cheap that every practitioner should, by purchasing both, be able to compare and decide for himself. The extensive circulation which this work has had for the last six years, is sufficient proof of the estimation in which it is held by the profession.

